# High quality draft genome sequence of *Mycoplasma testudineum* strain BH29^T^, isolated from the respiratory tract of a desert tortoise

**DOI:** 10.1186/s40793-018-0309-z

**Published:** 2018-04-11

**Authors:** Chava L. Weitzman, Richard L. Tillett, Franziska C. Sandmeier, C. Richard Tracy, David Alvarez-Ponce

**Affiliations:** 1 Biology Department, University of Nevada, Reno, USA; 2 Nevada Center for Bioinformatics, University of Nevada, Reno, USA; 3 Biology Department, Colorado State University, Pueblo, USA

**Keywords:** *Mycoplasma testudineum*, Desert tortoise, *Gopherus*, Upper respiratory tract disease, URTD

## Abstract

*Mycoplasma testudineum* is one of the pathogens that can cause upper respiratory tract disease in desert tortoises, *Gopherus agassizii*. We sequenced the genome of *M. testudineum* BH29^T^ (ATCC 700618^T^ = MCCM 03231^T^), isolated from the upper respiratory tract of a Mojave desert tortoise with upper respiratory tract disease. The sequenced draft genome, organized in 25 scaffolds, has a length of 960,895 bp and a G + C content of 27.54%. A total of 788 protein-coding sequences, six pseudogenes and 35 RNA genes were identified. The potential presence of cytadhesin-encoding genes is investigated. This genome will enable comparative genomic studies to help understand the molecular bases of the pathogenicity of this and other *Mycoplasma* species.

## Introduction

Species of the genus *Mycoplasma* have extremely small genomes, likely contributing to the need of the species to gain resources from host cells, and while *Mycoplasma* form a variety of relationships with hosts, many are pathogenic in vertebrates [1]. In North American tortoises, an upper respiratory tract disease is associated with both *Mycoplasma testudineum* and its close relative, *Mycoplasma agassizii* [2–5]. North American tortoise populations are in decline, with infectious disease as a possible agent in these declines [6–8], though importantly, our knowledge of the mechanisms of disease progression and its impacts on populations is lacking [9, 10]. To understand URTD, we must improve our understanding of the pathogens associated with the disease. By sequencing the genome of *M. testudineum*, we may gain insight into proteins associated with its pathogenicity and virulence.

Until now, DNA sequence data available for this species in GenBank was limited to ribosomal RNA genes and the associated intergenic spacer region, as well as the RNA polymerase beta subunit gene. To obtain genomic data on the species, we extracted DNA from a culture of the type-strain, BH29^T^, which was collected from the upper respiratory tract of a wild Mojave desert tortoise, *Gopherus agassizii* [3]. This sequencing work is part of a larger project addressing mycoplasmal variation among host species.

## Organism information

### Classification and features

*M. testudineum* infects the upper respiratory tracts of tortoises causing upper respiratory tract disease [3, 4]; however, recent investigations in wild tortoises suggest it may be present in the host without pathogenicity [11]. This microbe has been found in five tortoise species inhabiting North America—*G. agassizii**, G. morafkai, G. evgoodei, G. berlandieri,* and *G. polyphemus* [3, 11–13]—and its presence has yet to be investigated in the sixth tortoise congener, *G. flavomarginatus* (located in north-central Mexico). From wild samples, there is some indication that *M. testudineum* may have a facilitative relationship with *M. agassizii* in tortoise hosts, but interactions with other community members are unknown [11].

**Fig. 1:**
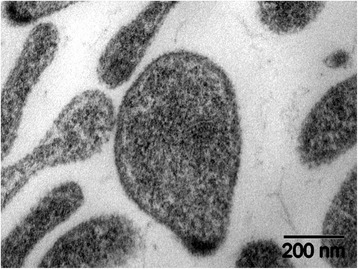
Transmission electron micrograph of thin section of *Mycoplasma testudineum* strain BH29^T^. Image from ref. [3], reproduced with permission from the publisher

*M. testudineum* is a sugar-fermenting, coccoid *Mycoplasma*, which is very similar in phenotype to the closely-related *M. agassizii* [3] (Table 1, Fig. 1). *M. testudineum* grows in culture at 22–30°C, with an optimal growth at 30°C [3] (Table 1). These temperatures are frequently experienced in their hosts during the seasons when tortoises are found to be most active [14, 15], though tortoise body temperatures can fluctuate well above or below these temperatures within a day and over the seasons [14–16].

**Table 1:** Classification and general features of *Mycoplasma testudineum* strain BH29^T^

MIGS ID	Property	Term	Evidence code^a^
	Classification	Domain *Bacteria*	TAS [48]
		Phylum “*Tenericutes*”	TAS [49]
		Class *Mollicutes*	TAS [50]
		Order *Mycoplasmatales*	TAS [51, 52]
		Family *Mycoplasmataceae*	TAS [52]
		Genus *Mycoplasma*	TAS [53, 54]
		Species *Mycoplasma testudineum*	TAS [3]
		Type strain: BH29^T^	
	Gram stain	Negative	TAS [3]
	Cell shape	Coccoid to pleomorphic	TAS [3]
	Motility	Non-motile	TAS [3]
	Sporulation	Nonspore-forming	NAS
	Temperature range	22–30 °C	TAS [3]
	Optimum temperature	30 °C	TAS [3]
	pH range; Optimum	Not reported	NAS
	Carbon source	Glucose, mannose, lactose, sucrose	TAS [3]
MIGS-6	Habitat	Tortoise respiratory tract	TAS [3]
MIGS-6.3	Salinity	Not reported	NAS
MIGS-22	Oxygen requirement	Aerobic	TAS [3]
MIGS-15	Biotic relationship	Symbiont	TAS [3]
MIGS-14	Pathogenicity	Pathogenic	TAS [3, 4]
MIGS-4	Geographic location	North America	TAS [3]
MIGS-5	Sample collection	1995	TAS [3, 23]
MIGS-4.1	Latitude	Not reported, BH29^T^ from Mojave Desert, USA	TAS [3]
MIGS-4.2	Longitude	N/A	NAS
MIGS-4.4	Altitude	N/A	NAS

^a^Evidence codes - *IDA* Inferred from Direct Assay, *TAS* Traceable Author Statement (i.e., a direct report exists in the literature), *NAS* Non-traceable Author Statement (i.e., not directly observed for the living, isolated sample, but based on a generally accepted property for the species, or anecdotal evidence). These evidence codes are from the Gene Ontology project [55]

**Fig. 2:**
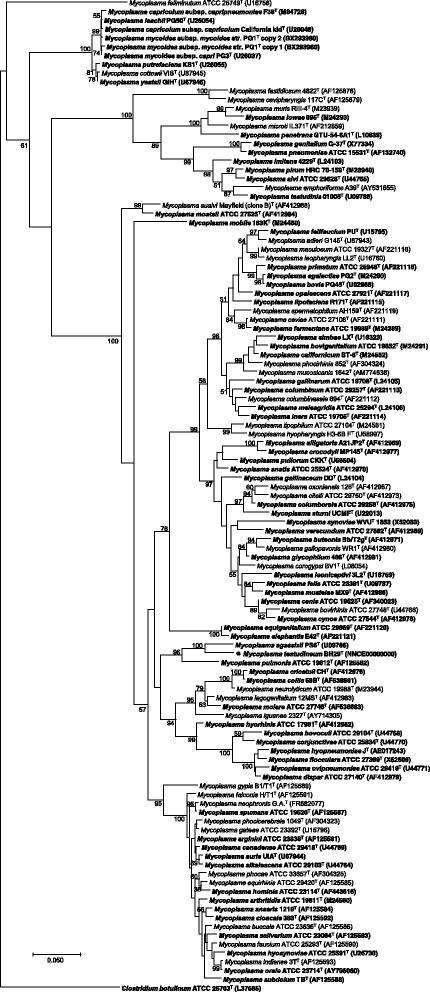
Phylogenetic tree of the *Mycoplasma* genus based on 16S rRNA gene sequences showing the phylogenetic position of *M. testudineum* BH29^T^ (●). All 16S sequences from the *Mycoplasma* genus were obtained from the SILVA database [17]. Only sequences in the ‘The All-Species Living Tree’ Project (LTP), release 128, were retained. This dataset only contains sequences from type strains, designated with a superscripted “T”. *Clostridium botulinum* strain ATCC 25763 was also included in the dataset as outgroup. Sequences were aligned using MUSCLE 3.8.31 [18]. A phylogenetic tree was obtained using the maximum likelihood method implemented in MEGA7 [19], with 1000 bootstrap replicates. Species with available genomes at the NCBI Genomes database or the Genomes Online Database are represented in bold face. GenBank accession numbers are shown in parentheses. Bootstrap support values above 50% are represented. The scale bar represents a divergence of 0.05 nucleotide substitutions per nucleotide position

To determine the placement of *M. testudineum* in the mycoplasmal phylogeny, all 16S rRNA gene sequences from the type strains of *Mycoplasma* species were obtained from the SILVA database [17] and aligned using MUSCLE 3.8.31 [18], and a phylogenetic tree was constructed using the maximum likelihood method implemented in MEGA7 [19] (Fig. 2). *M. agassizii* is a sister group of *M. testudineum* in the resultant tree, and the *M. testudineum*/*M. agassizii* clade is a sister group of *Mycoplasma pulmonis*—the agent of murine respiratory mycoplasmosis, which also seems to be present in humans who are in contact with rodents [20]. All three species fall within the hominis group of *Mycoplasma* (see ref. [21] for group definitions). The *M. testudineum* 16S rRNA gene sequence is 93.1 and 89.2% identical to those of *M. agassizii* and *M. pulmonis*, respectively. Remarkably, these species are not closely related to *Mycoplasma testudinis*, isolated from the cloaca of a spur-thighed tortoise (*Testudo graeca*) in the UK [22], which are placed in the pneumoniae group. A previous taxonomic analysis placed *M. testudinis* within the pneumoniae group (in agreement with our results), but placed *M. testudineum* and *M. agassizii* in different hominis subgroups: the hyorhinis and the fermentans groups, respectively [23]. Our result is, however, in agreement with that by Volokhov et al. [24], which was also based on 16S rRNA data.

## Genome sequencing information

### Genome project history

The type strain of *M. testudineum*, strain BH29^T^, was selected for sequencing. This strain was isolated from a nasal flush of the choana of a Mojave desert tortoise, which was filtered through a 0.45 μm filter and then grown in SP4 broth [2, 3]. Sequencing was conducted in October 2016. The Whole Genome Shotgun project was deposited at DDBJ/ENA/GenBank under the accession number NNCE00000000. The version described in this paper is the first version, NNCE01000000. A summary of the project information in compliance with MIGS version 2.0 [25] is shown in Table 2.

**Table 2:** Project information

MIGS ID	Property	Term
MIGS-31	Finishing quality	High quality draft
MIGS-28	Libraries used	Illumina Nextera XT
MIGS-29	Sequencing platforms	Illumina NextSeq500
MIGS-31.2	Fold coverage	64 ×
MIGS-30	Assemblers	SPAdes 3.10.1
MIGS-32	Gene calling method	NCBI Prokaryotic Genome Annotation Pipeline 4.2
	Locus Tag	CG473
	GenBank ID	NNCE00000000
	GenBank Date of Release	August 10, 2017
	GOLD ID	Gp0223607
	BIOPROJECT	PRJNA394956
MIGS-13	Source Material Identifier	ATCC 700618
	Project relevance	Animal parasite

### Growth conditions and genomic DNA preparation

Freeze-dried *M. testudineum*, strain BH29^T^, was obtained from the ATCC in November 2014 (ATCC 700618^T^) and had been cultured by the ATCC on *Spiroplasma* SP4 medium at 30°C in aerobic conditions. Genomic DNA was extracted using the Qiagen DNeasy Blood and Tissue Kit protocol for Gram-negative bacteria and eluted with ultra-pure water. Extracted DNA was quantified on a Qiagen QIAxpert system and with Picogreen analysis.

### Genome sequencing and assembly

Genome sequencing was conducted using the Illumina Nextera XT DNA Library Preparation Kit (Illumina, Inc., San Diego, USA) with the Illumina NextSeq500 platform (150 bp, paired-end) and 2 ng of starting genomic DNA at the Nevada Genomics Center (University of Nevada, Reno). Sequencing was performed in multiplex with multiple samples, using dual index sequences from the Illumina Nextera XT Index Kit, v2 (index 1, N701; index 2, S502). A total of 455,422 read pairs were obtained. Using Trimmomatic, version 0.36 [26], reads were trimmed to remove Nextera adapter sequences and low quality nucleotides from either end (average Phred score Q ≤ 5, four bp sliding window), and sequences trimmed to < 35 bp were removed. After trimming, 412,763 read pairs and 36,907 single-reads (the pairs of which were removed) remained. De novo genome assembly was performed using SPAdes 3.10.1 [27], using as inputs the trimmed paired reads, and the trimmed single reads (assembly k-mer sizes 21, 33, 55, and 77; with read error-correction enabled and ‘--careful’ mode mismatch correction). After removing scaffolds of less than 500 bp, the final assembly consisted of 25 scaffolds with a total length of 960,895 bp, an average length of 38,435 bp, and an N50 of 130,815 bp. The coverage was 64×.

### Genome annotation

Gene prediction was carried out using the NCBI Prokaryotic Genome Annotation Pipeline (PGAP) 4.2 [28]. For each predicted protein, (*i*) families were identified using the Pfam 31.0 [29] batch search tool (“gathering threshold” option), (*ii*) COG categories were assigned using eggNOG-mapper [30] based on eggNOG 4.5.1 data [31], (*iii*) signal peptides were identified using the SignalP server 4.1 [32], and (*iv*) transmembrane helices were inferred using the TMHMM server v. 2.0 [33]. CRISPR repeats were identified using PGAP and CRISPRFinder [34].

## Genome properties

The properties of the draft genome are summarized in Table 3. The final assembly consisted of 25 scaffolds, with a total length of 960,895 bp and a G + C content of 27.54%. The small genome size and low G + C content is consistent with those of other *Mycoplasma* genomes sequenced [35, 36]. PGAP [28] identified a total of 788 protein-coding genes, 6 pseudogenes, and 35 RNA genes. The identified RNA genes include 3 rRNAs (one 5S, one 16S and one 23S), 3 ncRNAs and 29 tRNAs. PGAP identified 4 CRISPR repeats, and CRISPRFinder [34] identified 4 “confirmed” repeats, and another 3 that were flagged as “questionable” by the server. The numbers of protein-coding genes in each COG category [37] are summarized in Table 4.

**Table 3:** Genome statistics

Attribute	Value	% of Total
Genome size (bp)	960,895	100.00
DNA coding (bp)	865,251^a^	90.05^c^
DNA G + C (bp)	264,678	27.54^c^
DNA scaffolds	25	100.00
Total genes	829	100.00
Protein coding genes	788	95.05^d^
RNA genes	35	4.22^d^
Pseudo genes	6	0.72^d^
Genes in internal clusters	–	–
Genes with function prediction	370^b^	46.95^e^
Genes assigned to COGs	539	68.40^e^
Genes with Pfam domains	558	70.81^e^
Genes with signal peptides	78	9.90^e^
Genes with transmembrane helices	217	27.54^e^
CRISPR repeats	4	–

^a^Protein-coding sequences, not including stop codons

^b^Proteins not annotated as “hypothetical protein” by PGAP

^c^Relative to genome size

^d^Relative to total number of genes

^e^Relative to protein-coding genes

**Table 4:** Number of genes associated with general COG functional categories

Code^a^	Value^b^	%age	Description
J	102	12.94	Translation, ribosomal structure and biogenesis
A	0	0.00	RNA processing and modification
K	19	2.41	Transcription
L	52	6.60	Replication, recombination and repair
B	0	0.00	Chromatin structure and dynamics
D	4	0.51	Cell cycle control, Cell division, chromosome partitioning
V	18	2.28	Defense mechanisms
T	4	0.51	Signal transduction mechanisms
M	8	1.02	Cell wall/membrane biogenesis
N	0	0.00	Cell motility
U	10	1.27	Intracellular trafficking and secretion
O	24	3.05	Posttranslational modification, protein turnover, chaperones
C	35	4.44	Energy production and conversion
G	66	8.38	Carbohydrate transport and metabolism
E	29	3.68	Amino acid transport and metabolism
F	25	3.17	Nucleotide transport and metabolism
H	14	1.78	Coenzyme transport and metabolism
I	8	1.02	Lipid transport and metabolism
P	37	4.70	Inorganic ion transport and metabolism
Q	1	0.13	Secondary metabolites biosynthesis, transport and catabolism
R	0	0.00	General function prediction only
S	89	11.29	Function unknown
–	249	31.60	Not in COGs

Percentages are based on the total number of protein coding genes in the genome

^a^COG category code

^b^Number of genes in the category

## Insights from the genome sequence

**Fig. 3:**
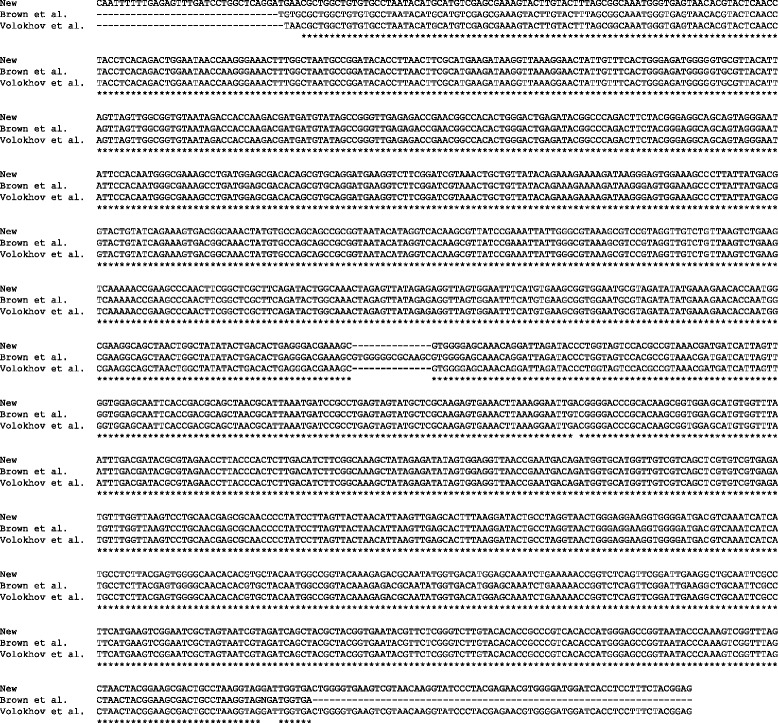
Comparison of the 16S rRNA gene sequences generated by Brown et al. [3], by Volokhov et al. [24], and in our study. All three sequences correspond to *M. testudineum* BH29^T^. Asterisks represent identical sites

Brown et al. [3] sequenced most of the 16S rRNA gene of *M. testudineum* strain BH29^T^ (GenBank ID: AY366210). They had previously sequenced the homologous region for *M. testudineum* strain H3110, which differed only in one nucleotide position (GenBank ID: U19768, ref. [23]). Comparison of their BH29^T^ sequence and that obtained by us revealed 5 point differences and an indel of 14 nucleotides (present in Brown et al.’s sequence but not in ours) (Fig. 3). Remarkably, 4 of the 5 point differences were located toward the ends of Brown et al.’s sequence, and thus may represent sequencing errors. The other differences probably represent mutations accumulated since the isolation of the strain in 1995. Our 16S rRNA gene sequence is identical to that generated by Volokhov et al. [24], with the exception of the first nucleotide of Volokhov et al.’s sequence. Nevertheless, the placement of *M. testudineum* in the tree (Fig. 2) is not affected by the particular sequence used.

In general, *Mycoplasma* cells need to adhere to mucosal epithelial cells of the hosts as a pre-requisite for pathogenesis. The mechanisms of adhesion are relatively well understood in *Mycoplasma pneumoniae* and its close relatives, but much less so in other *Mycoplasma* groups [38]. We used BLASTP and TBLASTN (*E* < 10^− 5^; low-complexity regions filtered out) to search for homologs of *M. pneumoniae* cytadhesins P1, P30, P65, P40 and P90 —proteins involved in adhesion— and cytadhesin accessory proteins Hmw1, Hmw2 and Hmw3 in all available *Mycoplasma* genomic data (nr database). We only found homologs in species closely related to *M. pneumoniae* (*Mycoplasma genitalium*, *Mycoplasma gallisepticum*, *Mycoplasma pirum*, *Mycoplasma alvi*, *Mycoplasma imitans*, and *M. testudinis*), as previously noted [38, 39]. Searches against the *M. testudineum* BH29^T^ proteome detected no hits, and none of the 788 predicted *M. testudineum* proteins contained any of the Pfam domains present in the *M. pneumoniae* cytadhesins and accessory proteins (domains “CytadhesinP1”, “Adhesin_P1”, “Cytadhesin_P30”, “MgpC” and “EAGR_box”). These observations may have at least three alternative explanations: (*i*) the adhesion proteins used by *M. pneumoniae* may be specific to its group, (*ii*) adhesion proteins evolve very fast, perhaps due to co-evolutionary races, thus hindering the detection of distant homologs, or (*iii*) *M. testudineum* may exhibit limited adhesion capabilities. In support of the first possibility, *M. pulmonis*, the most closely related species to the *M. testudineum*/*M. agassizii* clade (Fig. 2), is known to have adhesion mechanisms different from *M. pneumoniae*: *M. pneumoniae* exhibits a specialized attachment organelle, whereas *M. pulmonis* adhesion takes place by generalized interaction of the pathogen and the host cell membranes [40]. The adhesins of *M. pulmonis* are unknown. In support of the second scenario, putative cytadhesins identified in *M. pirum* and *M. gallisepticum* are only 26–29% identical at the amino acid level to those of *M. pneumoniae* [41, 42].

To extend our search, we obtained a list of known *Mycoplasma* adhesins from the UniProt database [43] (search: “*Mycoplasma* adhesin”). Again, BLASTP and TBLASTN searches (*E* < 10^− 5^; low-complexity regions filtered out) against the *M. testudineum* BH29^T^ proteome/genome did not identify any significant hits. *M. pneumoniae* proteins GAPDH and EF-Tu and *M. hominis* protein OppA have been reported to be adhesins in addition to their traditional functions [44–46]. We found homologs of all three proteins in *M. testudineum*. It should be noted, however, that this does not guarantee that these proteins act as adhesins in *M. testudineum*. For instance, whereas *M. pneumoniae* EF-Tu binds fibronectin [45], *M. genitalium* EF-Tu, which is 96% identical, does not [47]. The *M. testudineum* protein is only 70% identical to that of *M. pneumoniae*, and serine 343, proline 345, and threonine 357 (replacement of which significantly reduces the fibronectin binding of EF-Tu in *M. pneumoniae*; ref. [47]) are not conserved in *M. testudineum*. Additional work will be required to understand the mechanisms of adhesion in *M. testudineum* and its close relatives.

## Conclusions

We have obtained a draft genome sequence of *M. testudineum* BH29^T^ isolated from the upper respiratory tract of a desert tortoise with URTD in the Mojave Desert. Our analysis revealed some features typical of *Mycoplasma* genomes: a very small size and low G + C content. The new genome will enable comparative genomic studies to help understand the molecular bases of the pathogenicity of this and other *Mycoplasma* species.

## Abbreviations

ATCC: American Type Culture Collection
BLAST: Basic local alignment search tool
COG: Clusters of Orthologous Groups
EF-Tu: Elongation factor Tu
GAPDH: Glyceraldehyde-3-phosphate dehydrogenase
MIGS: Minimum information on the genome sequence
NCBI: National Center for Biotechnology Information
OppA: Substrate-binding domain of the oligopeptide permease
